# Antivirals to prepare for surges in influenza cases: an economic evaluation of baloxavir marboxil for the Netherlands

**DOI:** 10.1007/s10198-024-01683-1

**Published:** 2024-03-14

**Authors:** Simon van der Pol, Maarten J. Postma, Cornelis Boersma

**Affiliations:** 1 Health-Ecore, Utrechtseweg 60, 3704 HE Zeist, The Netherlands; 2 Department of Health Sciences, University of Groningen, University Medical Center Groningen, Groningen, The Netherlands; 3 Department of Economics, Econometrics & Finance, University of Groningen, Groningen, The Netherlands; 4 Department of Management Sciences, Open University, Heerlen, The Netherlands

**Keywords:** Baloxavir, Antiviral, Seasonal influenza, Cost effectiveness, Budget impact, C31, I13

## Abstract

**Objectives:**

We perform a cost-effectiveness analysis (CEA) and budget impact analysis (BIA) of baloxavir marboxil compared to current care in the Netherlands for patients at risk of influenza-related complications, including patients with comorbidities and the elderly.

**Methods:**

In the CEA, a decision tree model was developed to assess the cost-effectiveness of baloxavir marboxil for a cohort of 52-year-olds from a societal perspective. A lifetime horizon was taken by incorporating the quality-adjusted life expectancy. The BIA included different epidemiological scenarios, estimating different plausible epidemiological scenarios for seasonal influenza considering the whole Dutch population with an increased risk of influenza complications.

**Results:**

The base-case ICER was estimated to be €8,300 per QALY. At the willingness-to-pay threshold of €20,000 per QALY, the probability of being cost effective was 58%. The base-case expected budget impact was €5.7 million on average per year, ranging from €1.5 million to €10.5 million based on the severity of the influenza epidemic and vaccine effectiveness.

**Conclusion:**

In the Netherlands, baloxavir is a cost-effective treatment option for seasonal influenza, with a base-case ICER of €8,300 per QALY for the population aged 60 years and over and patients at high risk of influenza-related complications. For a large part, this ICER is driven by the reduction of the illness duration of influenza and productivity gains in the working population.

**Supplementary Information:**

The online version contains supplementary material available at 10.1007/s10198-024-01683-1.

## Introduction

Influenza is an acute respiratory infection caused by various types of influenza A and B viruses in humans [1]. In many cases an infection with influenza will not lead to severe illness: these patients will not seek care and the infection will be self-limiting [2]. However, for a small percentage of patients an infection causes severe disease and these patients may need to be admitted to hospital [3]. A complicated illness course may be caused by a co-infection with bacteria, which can lead to a pneumonia, acute bronchitis or myocarditis [4]. Most cases of influenza will consistently be during the Winter season, but the intensity of epidemics vary considerably throughout the years [5]. The number of consults in Dutch primary care for influenza-like illness (ILI) differed considerably in the period between October 2013 and May 2019: ranging from 1200 consults during the influenza season to 2400 consults per 100,000 inhabitants [5]. The number of influenza-related hospitalizations also varies, ranging from 650 in 2014 to 9300 in 2018 (ICD J09–J11) [6]. The number of reported influenza-related deaths in this period ranged from 67 to 1207 [7], although this probably is an underestimation as not all influenza-related mortality will be primarily recorded as related to influenza. Excess deaths during severe epidemics can be as high as 9444, as was the case for the influenza season of 2017–2018 [8]. The majority of patients hospitalized and dying from influenza are over 65 years old [7]. Vaccinations have played an important role in preventing morbidity and mortality related to influenza, and have previously been estimated to reduce hospitalizations with 24% and deaths with 35% in the Netherlands [9].

In the Netherlands, elderly aged 60 years and older are invited for an annual influenza vaccination which can be administered by the general practitioner (GP) [10]. In addition, patients with certain comorbidities, such as dementia, obesity, and COPD/asthma, are also eligible for influenza vaccination [10]. Attaining a high coverage of influenza vaccinations will be important to be prepared for a potential influenza epidemic, especially in older adults. However, not everyone is willing to get the vaccine and efficacy is non-optimal as the strains against which the vaccine protects may not always match with the circulating influenza strains. In addition, on the national policy level, there is no target for the influenza vaccination coverage and for at-risk populations getting vaccinated remains highly facultative. Here, the Dutch government aims at good communication about the national vaccination programme for influenza, rather than implementing performance indicators such as vaccination coverage.

Antiviral treatment may be an important addition to the control of influenza infection and its complications to combat surges of influenza, especially in a world where the available hospital capacity may be under high pressure during the respiratory season due to COVID-19, influenza, and other (co-)infections. However, the use of antiviral treatment against influenza is yet uncommon in the Netherlands. One of the most common treatments is oseltamivir, but this is not reimbursed in the Netherlands [11] and the use of antivirals is currently not recommended by the Dutch society of General Practice (NHG) [12].

Recently, a new antiviral treatment was introduced: baloxavir marboxil (baloxavir further in text), also known under the brand name Xofluza®. In the CAPSTONE 1 and -2 trials, this antiviral was more effective at reducing the duration of symptoms and reduced influenza-related complications in otherwise-healthy adults and patients at high risk of complications compared to placebo [13, 14]. The effectiveness in alleviating influenza symptoms of baloxavir was similar to oseltamivir [13, 14], but baloxavir was more effective at reducing the viral load of influenza and the time to cessation of viral shedding [13], which may reduce transmission between patients [15]. Baloxavir needs to be taken within 48 h after the onset of symptoms and only needs to be administered once [14]. This treatment may be a good alternative not only for unvaccinated patients at risk of influenza-related complications, such as the older adults and patients with comorbidities but also for vaccinated patients during influenza seasons in which the vaccine is reported to be less effective by the Dutch public health institute due to a mismatch with circulating influenza strains. Recently, it was decided to not reimburse baloxavir in the Netherlands, however, the cost-effectiveness was not considered in this decision [16].

In this paper, we will perform a cost-effectiveness analysis (CEA) and budget impact (BI) analysis of baloxavir compared to current care in the Netherlands for patients at risk of influenza-related complications, including patients with comorbidities and the elderly. We will assess different scenarios regarding influenza incidence, vaccination effectiveness, and vaccination coverage, as these are highly uncertain after two years of strict infection prevention and control measures during the pandemic.

## Methods

### Intervention

With this analysis, we compared the current standard of care for seasonal influenza patients in the Dutch outpatient setting, to a scenario where baloxavir is considered for patients that have an indication for the annual influenza vaccination. The current standard of care can be described as symptomatic treatment, primarily with paracetamol, as the use of antivirals is not currently recommended in the Netherlands [12]. As the Netherlands has historically been conservative regarding the use of influenza treatments, we did not consider the introduction for the otherwise-healthy population under 60 years. In addition, since baloxavir was not approved in Europe for children under 12 years at the time of writing, we also did not consider this group.

### Cost-effectiveness model

**Fig. 1:**
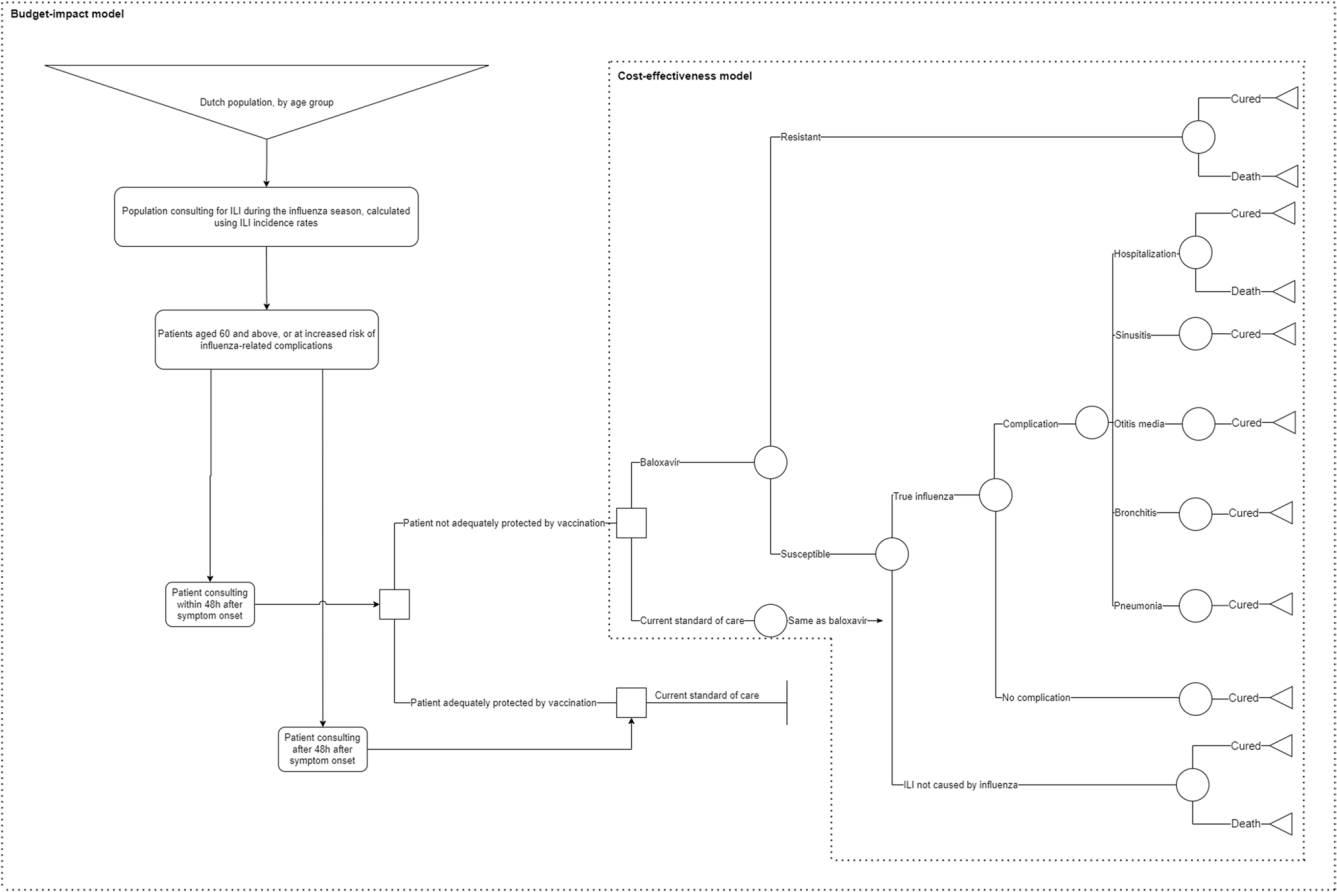
Overview of cost-effectiveness and budget impact models—*ILI* influenza-like illness

A probabilistic static closed cohort model with a typical short time frame was developed, as most influenza episodes will be short and without long-term complications. A lifetime horizon was taken by incorporating the quality-adjusted life years (QALYs) lost in the case of mortality, considering the cohort’s life expectancy. In line with Dutch guidelines, costs were discounted with 4% and health losses with 1.5% [17]. An overview of the model, visualized as a decision tree, is provided in Fig. 1.

For the base-case CEA, the target population was chosen to reflect the CAPSTONE-2 trial as closely as possible: a cohort of 1000 52-year-olds was considered, an even mix between males and females and all with underlying comorbidities [14]. Different age groups were considered in separate scenarios. The analysis was conducted from the societal perspective, according to the guidelines for conducting CEA in the Netherlands [17]. The model is available upon reasonable request. The Consolidated Health Economic Evaluation Reporting Standards checklist was included in the supplementary information [18].

### BI model

A separate model, also displayed in Fig. 1, was developed to assess the BI of baloxavir over five influenza seasons starting in October 2022 and ending in May 2028. The BI was calculated for the whole eligible population in the Netherlands, which was estimated to be around 5.6 million individuals in the Netherlands in 2022, covering both vaccinated and unvaccinated people [19, 20]. The healthcare perspective was used for the BI analysis, e.g. no productivity losses were included.

For the BI model, the annual number of patients eligible for a baloxavir prescription needed to be estimated. ILI incidence as published by the World Health Organization for the Netherlands was used [5], using the five most recent influenza seasons before the COVID-19 pandemic. The influenza season was assumed to start every year in October and end in May. Baloxavir is only indicated for patients consulting their GP within 48 h after symptom onset, which was estimated to be the case for 67% of patients [21]. For the decision to prescribe baloxavir, we considered the patient’s vaccination status and the protection of the vaccine to the circulating strain of influenza. In the intervention group, all unvaccinated patients eligible for the annual influenza vaccine consulting for ILI would be prescribed baloxavir by the GP, while vaccinated patients would only be prescribed baloxavir if the vaccine was a mismatch with the circulating strains of influenza. To assess the proportion of patients adequately protected by an influenza vaccine, Dutch vaccine coverage data were used [20]. The key parameters for the BI analysis are included in the supplementary information.

### Input parameters

#### Clinical inputs

The main clinical inputs considered were the duration of illness and mortality. 38% of patients consulting for ILI were considered to have an infection with influenza and baloxavir was assumed to only be effective for the “true influenza” group [22]. Since this varies highly between influenza seasons, this number was extensively varied in the probabilistic sensitivity analysis (PSA) [22]. Currently, influenza point-of-care (POC) diagnostics are not recommended in the Netherlands [4, 23]. To incorporate a potential scenario where POC diagnostics for influenza are widely implemented, a scenario was included where only patients with true influenza were treated with baloxavir. For patients with true influenza the duration of illness was modelled using data from the CAPSTONE-2 trial. The secondary clinical endpoint “time to return to pre-influenza health” was used, as this was expected to capture the full extent of the patients’ disease episode [14]. For the baloxavir group, this corresponded to an average duration of illness of 5.3 days, while this was 6.2 for the current standard of care group [14]. As a scenario, the primary efficacy endpoint was used: “time to improvement of symptoms” [14]. Various complications were included using the data reported in the CAPSTONE-2 trial: hospitalization, sinusitis, otitis media, bronchitis, and outpatient pneumonia [14]. Mortality was assumed to only occur in the hospital. Previously-reported in-hospital mortality stratified for age groups were used: 4% for 15–64 year olds (base case) and 43% for patients older than 65 years [24].

In case of an influenza infection resistant against baloxavir or ILI caused by other pathogens, simplified inputs were used as the outcomes were assumed to be the same for the baloxavir and current standard of care arms in the model. The disease duration for this group was 7.7 days in the base case [21] and the mortality rate 0.1% for patients aged 50–64 (base case) or 1.4% for patients aged 65 years and older [25]. Although baloxavir resistance was not considered in the base case, we did include it as a scenario. The main clinical inputs for the model are displayed in Table 1.

**Table 1:** Cinical input parameters

Input	Baloxavir arm	Current care arm	Distribution	References
General probabilities				
True influenza cases	38% (lower bound: 12%, upper bound: 65%)		Beta PERT	[22]
Baloxavir resistance	0%		None	Assumption
Duration of illness (days)				
Illness days before consulting	1 day		None	Assumption
Duration of illness (true influenza)	Time to return to pre-influenza health: 5.3 days (CI 4.4–6.4) Time to improvement of symptoms: 3.1 days (CI 2.8–3.5)	Time to return to pre-influenza health: 6.2 days (CI 5.3–7.3) Time to improvement of symptoms: 4.3 days (CI 3.9–4.7)	Gamma	[14]
Duration of illness (ILI caused by other pathogens)	7.7 days (CI 7.0–8.5)		Gamma	[21]
Duration of outpatient complications	Otitis media: 9.4 days (CI 3.5–15.2) Other: 10.7 days (CI 4.8–16.5)		Gamma	[25]
Duration of hospitalization	5 days (lower quartile: 3, upper quartile: 11)		Gamma	[29]
Complications				
Hospitalization	0.8% (alpha: 3)	1.3% (alpha: 5)	Dirichlet	[14]
Sinusitis	0.3% (alpha: 1)	2.1% (alpha: 8)	Dirichlet	[14]
Otitis media	0% (alpha: 0)	0.8% (alpha: 3)	Dirichlet	[14]
Bronchitis	1.8% (alpha: 7)	6.0% (alpha: 23)	Dirichlet	[14]
Outpatient pneumonia	0% (alpha: 0)	0.8% (alpha: 3)	Dirichlet	[14]
No complication	97.1% (alpha: 377)	89.0% (alpha: 347)	Dirichlet	[14]
Mortality after influenza-related hospitalization	4% (3.7–4.3)		Beta	[24]
Mortality non-influenza ILI episode	0.1%		None	[25]
Quality of life				
Disutility ILI	0.32 (0.31–0.33)		Beta	[26, 27]
Outpatient complications	0.37 (0.27–0.47)		Beta	[25]
Inpatient complications	0.37 (0.27–0.47)		Beta	[25]
Life expectancy				
Life expectancy^a^	31.3 years		None	[7]
Quality-adjusted life expectancy^a^ (discounted)	22.1 years		None	[7, 28]

^*a*^*indicates an age-dependent variable*

#### Utilities

For both true influenza and ILI caused by other pathogens, a disutility of 0.32 quality-adjusted life days was used per day of illness [26, 27]. Considering a full influenza episode, this resulted in a per-patient QALY loss of 0.0046 and 0.0055 QALYs in the baloxavir and current standard of care groups, respectively [14]. For both inpatient and outpatient complications, a disutility of 0.37 quality-adjusted life days was considered [25]. These disabilities were based on Belgian and British data, which was assumed to be comparable to the Dutch setting and varied in sensitivity analyses. For deceased patients, the QALYs lost were calculated using the age and sex-dependent life expectancy, which was 27.4 QALYs in the base case. Age and sex-dependent utilities were also applied [28] and future QALYs were discounted.

Table 1* clinical input parameters*

#### Costs

The main costs are displayed in Table 2. The costs were based on Dutch reference prices whenever possible. Hospitalization costs for influenza-related complications were used as published previously [29], as well as costs associated with outpatient pneumonia [30]. For the other outpatient complications, the costs were assumed equal to one GP consult. Productivity losses were included in the model, based on an average number of hours worked per day of 4.4 [31], an unemployment rate of 14% [32], and hourly wages of €36.19 [33]. For unpaid work, people were assumed to spend an average of 4 h per week on this [34] and it was valued as informal care at €14.58 per hour [33]. In case of mortality, the production losses were estimated using the friction cost method, with a friction period of 126 days [17, 33, 35, 36]. Future non-related medical costs for all survivors were included in a scenario analysis using the PAID 3.0 tool [37, 38]. All costs were inflated to 2021 euros using the consumer price index [39].

**Table 2:** Cost inputs

Item	Unit costs	References
Baloxavir, including pharmacy fee	€133	[11]
GP consult	€34.36	[33]
Hospitalization	€7,340.07	[29]
Outpatient pneumonia	€647.46	[30]
Average productivity losses per day^a^	€138.42	[31–33]
Productivity losses per death^a^	€11,765.58	[31–33]
Average productivity losses for unpaid work per day	€8.33	[33, 34]
Productivity losses per death for unpaid work^a^	€708.08	[33, 34]
Indirect medical costs surviving patients^a^	€134,875	[37, 38]

^*a*^*indicates an age-dependent variable*

### Analysis

#### Cost-effectiveness analysis

The main health-economic outcome considered was the incremental cost-effectiveness ratio (ICER) expressed as the costs per QALY. A deterministic sensitivity analysis was performed to assess the effect of varying individual input parameters on the ICER, and this was visualized using a Tornado diagram. The inputs were varied with plus and minus 25%. A PSA was performed to assess the effect of varying all parameters simultaneously in a Monte Carlo simulation using 10,000 model replications, using the distributions and ranges of the input parameters as detailed in Table 1. A cost-effectiveness plane and cost-effectiveness acceptability curve (CEAC) were constructed to visualize the results. Various scenario analyses were performed:

- outcome used is “Time to improvement of symptoms” from CAPSTONE-2;
- only patients with true influenza are treated with baloxavir;
- baloxavir resistance set to 10% (assumption);
- cohort of age 65;
- cohort of age 75;
- inclusion of indirect medical costs in life years gained;
- using a healthcare perspective;
- using a healthcare perspective and equal discounting at 3.5%.

For these scenarios the deterministic ICER was calculated, as well as the probability of being cost-effective at both the €20,000 and the €50,000 willingness-to-pay thresholds, which are used in the Netherlands. All ICERs were rounded to the nearest hundreds of euros.

##### BI

The BI was expected to vary depending on the incidence of ILI and the effectiveness of the influenza vaccination campaign. In the base case, we estimated the average BI of baloxavir by using historical incidence data over several influenza seasons and averaging these. In addition, we included two sensitivity analyses: a best-case scenario with low incidence of ILI and a high vaccination coverage, taking the COVID-19 booster campaign as an example, and a worst-case scenario where a mismatch of the vaccine with the currently circulating virus was assumed and all patients consulting for ILI aged 60 years or within a risk group were treated with baloxavir.

## Results

### Cost effectiveness

**Fig. 2:**
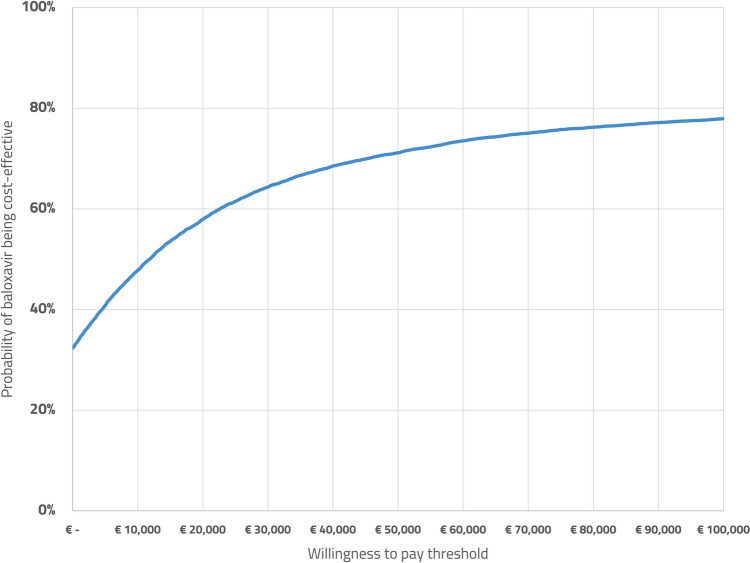
Cost-effectiveness acceptability curve of baloxavir compared to care as usual

**Fig. 3:**
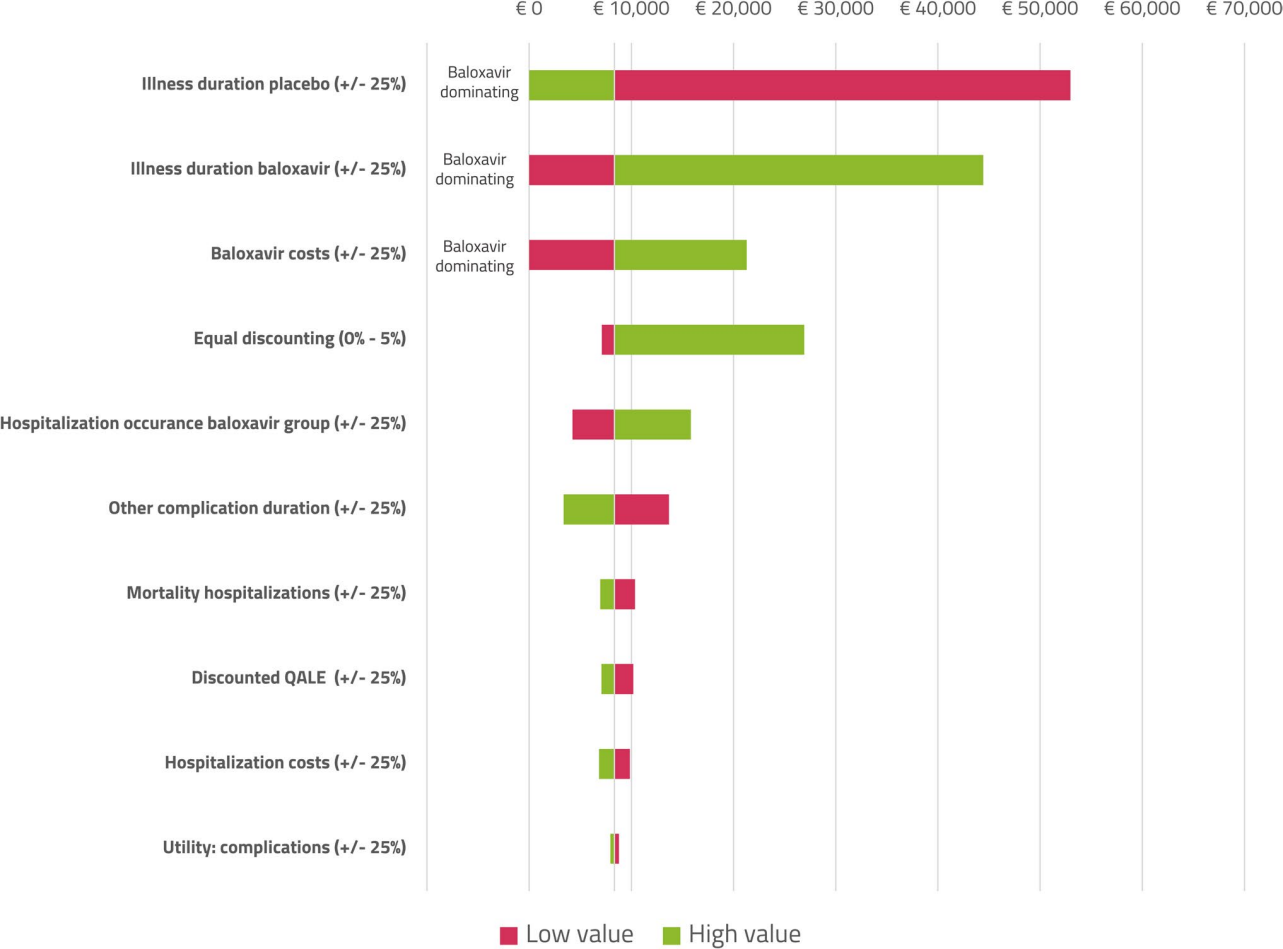
Tornado diagram of deterministic sensitivity analysis

The base-case ICER was estimated to be €8,300 per QALY, see also Table 3 for an overview. At the willingness-to-pay threshold of €20,000 per QALY, the probability of being cost effective was 58%, or 71% at 50,000 per QALY, see also Fig. 2 for the CEAC and the CE plane included in the supplementary information. Table 3 also includes various scenario analyses. The DSA is shown in Fig. 3; the main parameters influencing the model are the illness duration, costs of baloxavir, discounting, hourly wages, and the occurrence of complications, including hospitalizations.

**Table 3:** Model results, QALYs and costs are reported per patient. QALYs are rounded to three decimals, costs are rounded to the nearest Euro, ICERs are rounded to the nearest hundreds of Euros

Scenario	QALYs lost	Costs	ICER	Probability cost effective at €20,000 per QALY
1. Base case	Baloxavir: 0.023 Standard-of-care: 0.025	Baloxavir: €1,347 Standard-0f-care: €1,334	€8,300	58%
2. “Time to improvement of symptoms” used as main outcome	Baloxavir: 0.023 Standard-of-care: 0.024	Baloxavir: €1,265 Standard-of-care: €1,260	€3,100	64%
3. 100% true influenza cases	Baloxavir: 0.017 Standard-of-care: 0.021	Baloxavir: €1,237 Standard-of-care: €1,346	Dominating	83%
4. 10% resistance against baloxavir	Baloxavir: 0.023 Standard-of-care: 0.025	Baloxavir: €1,339 Standard-of-care: €1,319	€14,700	51%
5. Cohort of age 65	Baloxavir: 0.179 Standard-of-care: 0.188	Baloxavir: €504 Standard-of-care: €443	€7,200	69%
6. Cohort of age 75	Baloxavir: 0.116 Standard-of-care: 0.121	Baloxavir: €228 Standard-of-care: €155	€13,300	57%
7. Inclusion of indirect medical costs in life years gained	Baloxavir: 0.023 Standard-of-care: 0.025	Baloxavir: €127,488 Standard-of-care: 127,469	€12,500	53%
8. Using a healthcare perspective	Baloxavir: 0.023 Standard-of-care: 0.025	Baloxavir: €137 Standard-of-care: €60	€50,100	14%
9. Using a healthcare perspective and an equal discounting rate of 3.5%	Baloxavir: 0.019 Standard-of-care: 0.021	Baloxavir: €137 Standard-of-care: €60	€59,900	10%

*QALY *Quality Adjusted Life Year

### BI

The base-case expected BI was €5.7 million on average per year. The best-case scenario, where ILI incidence was assumed to be at the lowest level since 2014–2015 with a good match between the vaccine and the circulating types of influenza, was €1.5 million. The worst-case, with a high incidence of ILI and a mismatch between the vaccine and circulating types of influenza, increased the BI to €10.5 million. A relevant outcome for the healthcare sector is the number of influenza-related hospitalizations, which is reduced annually by 263 in the base-case scenario and up to 501 in the worst-case scenario. A full overview is displayed in Table 4.

**Table 4:** Average annual budget impact of baloxavir over 5 years

Scenario	Incidence of influenza-like illness (annual average)	Vaccination coverage and effectiveness	Eligible consultations for baloxavir (annual average)	Baloxavir costs (annual average)	Total prevented hospitalizations	Savings due to prevented complications	Incremental budget impact
Base case	382,737	Based on historical data, 2014–2019[20, 47–51]	60,557	€8,054,092	263	€2,370,116	€5,683,977
Best case	295,252	Based on vaccination coverage first booster COVID-19, match of vaccine with circulating influenza	16,089	€2,139,859	72	€644,094	€1,495,765
Worst case	475,808	Mismatch of vaccine with circulating influenza, vaccination coverage not regarded	112,516	€14,964,637	501	€4,513,311	€10,451,326

## Discussion

With a base-case ICER of €8,300 per QALY, baloxavir is likely to be cost effective for the Dutch setting, although the probabilistic results show quite some uncertainty around this estimate. This uncertainty is mainly due to the small number of complications measures in the CAPSTONE 2 trial, and the resulting uncertainty. In around 20% of the simulations, baloxavir is associated with health losses, i.e. the ICER is in the upper-left quadrant. In those simulations, the probability of some of the complications, mainly the number of hospitalizations is higher in the baloxavir arm. Baloxavir is considered cost-saving in around 30% of the simulations: the productivity gains and prevented complications are sufficient to offset the costs of baloxavir. We have included several scenarios for older adult populations, and both the deterministic and probabilistic results are robust, i.e., baloxavir can be considered cost-effective. Productivity losses play a minor role in these older cohorts, but the cost effectiveness is mainly driven by prevented hospitalizations and mortality, as older generations have higher risks for these clinical events.

The BI of baloxavir is estimated at €5.7 million per year in the base-case scenario and mainly depends on the incidence of ILI, the vaccine effectiveness, and the vaccine coverage. Between the best-case and the worst-case scenarios, the BI ranged from €1.5 million to €10.5 million.

This is the first study assessing the cost-effectiveness and BI for baloxavir in a European country. Two economic analyses have been published for Japan [40, 41], one of which [40] assessed baloxavir for high-risk patients. Although the authors also used a decision tree, there are some important differences due to the setting for which the cost-effectiveness was assessed: in the Japanese study baloxavir was compared to laninamivir, the most-often prescribed antiviral for influenza in that market, and the model incorporated the use of a rapid diagnostic test for influenza [40]. No productivity losses were incorporated in the Japanese study [40]. However, since the QALY differences between the comparators are quite small, differences in drug costs and model assumptions can have a major impact on the ICER as can be seen in the deterministic sensitivity and scenario analyses. For other countries, other assumptions and costs inputs may need to be made. An example of a major difference could be that not in all countries, productivity losses are included, as is the case for the Netherlands [17]. The exclusion of productivity losses increases the ICER to €50,100 per QALY. Most countries use equal discounting rates for costs and effects, which means that our final scenario, in which an equal discounting rate was used and productivity losses were excluded may be the most generalizable result for other European countries. In this scenario the deterministic ICER was €59,900 per QALY. In most European countries, alternative influenza antiviral treatment is not often prescribed [42], but for the countries where this is the case, another comparator may be more appropriate.

In a previous study, we showed the potential value of improved diagnostics for respiratory-tract infections, including ILI, in Dutch primary care [43]. Although improved diagnostic strategies had the potential to reduce antimicrobial resistance, significant investments needed to be made in diagnostics [43]. The scenario included in the present study where the proportion of true influenza cases is increased from 38 to 100% shows that this makes baloxavir treatment highly likely to be cost saving. Improved influenza diagnostics could further personalize baloxavir prescriptions, as primarily patients likely to benefit from the treatment will receive it. This study shows that further targeted treatment could offset some of the additional costs of improved diagnostic strategies, provided adequately performing diagnostic tests are available.

The present study has some limitations. The modelled results are uncertain, due to the uncertainty associated with the main outcomes, such as most importantly the hospitalization rate. As influenza only causes serious complications in a small fraction of infected patients [14], a large number of patients would need to be included in a trial to show a significant difference in all the separately modelled complications. However, the aggregated occurrence of influenza-associated complications was significantly lower in the baloxavir group compared to placebo [14]. There were no adverse events included in the model, as the safety profile of baloxavir in adults is very similar to placebo [13, 14, 44]. We only included inpatient mortality, which we expect to underestimate the real mortality as patients may also decease in the outpatient setting. Although baloxavir has shown to reduce the viral load of influenza and the time to cessation of viral shedding, which may impact transmission [13, 15], this was not included in the CEA. We do not expect the targeted implementation of baloxavir, as proposed in this paper, would have a major impact on influenza transmission. Finally, we assessed a seasonal influenza situation, and although baloxavir may provide value to prepare for influenza pandemics as well, this was outside of the scope of the current research.

As we enter a situation where further measures to reduce COVID-19 transmission are hopefully not necessary, a surge of other respiratory infections is something that should be considered by policy makers. This includes a toolbox with adequate vaccination programmes, sufficient diagnostic capacity, and effective treatment against infections. For the Netherlands, baloxavir is a cost-effective influenza antiviral treatment that can be a part of a comprehensive strategy to manage the effects of respiratory infections in terms of healthcare capacity and the economic effects of absence from work. Therefore, in the likely situation of a seasonal influenza outbreak, not reimbursing baloxavir could be considered a loss of opportunity from health, broader societal and economic perspectives. Yet, the interdependency between seasonal risk, effectiveness of the seasonal vaccine and vaccine coverage clearly defines the complexity of assessing the added value and need for antivirals in the treatment of influenza. Although an effective influenza vaccination programme should remain a priority, antiviral treatment can be important from an equity perspective, as vaccine coverage in the Netherlands is lower in groups with a low socio-economic status [45] and certain religious subgroups [46].

## Conclusion

In the Netherlands, baloxavir is a cost-effective treatment option for seasonal influenza, with a base-case ICER of €8,300 per QALY for the population aged 60 years and over and patients at high risk of influenza-related complications. For a large part, this ICER is driven by the illness duration of influenza and productivity gains in the working population.

## Supplementary Information

Below is the link to the electronic supplementary material.

Supplementary file1 (PDF 1779 KB)

## Data Availability

The data that support the findings of this study are available from the corresponding author, upon reasonable request.
