# Supplementary material for: Antivirals to prepare for surges in influenza cases: an economic evaluation of baloxavir marboxil for the Netherlands
Source: Eur J Health Econ. 2024 Mar 14;25(9):1557–67. doi: 10.1007/s10198-024-01683-1 (PMC11512865; doi:10.1007/s10198-024-01683-1)
Supplement: Supplementary file 1 — Supplementary file1 (PDF 1779 KB) [file 10198_2024_1683_MOESM1_ESM.pdf]

HEALTH-ECORE
For sustainable impact

Supplementary material

Antivirals to Prepare for Surges in Influenza Cases

An Economic Evaluation of Baloxavir Marboxil for the Netherlands

Simon van der Pol, Maarten J. Postma, Cornelis Boersma

Contact: simonvanderpol@health-ecore.com

Contents

CHEERS checklist 2
Key parameters budget impact analysis 5
Cost-effectiveness plane base-case analysis 6
Extended results scenario analyses cost-effectiveness 8
Scenario 1 (base case) 8
Scenario 2 9
Scenario 3 10
Scenario 4 11
Scenario 5 12
Scenario 6 13
Scenario 7 14
Scenario 8 15
Scenario 9 16
References 17

CHEERS checklist

The following table was adapted from Husereau *et al.* 2022 [1]. The budget impact analysis is outside the scope of the CHEERS checklist.

Section/topic	Item No	Guidance for reporting	Reported in section
Title			
Title	1	Identify the study as an economic evaluation and specify the interventions being compared	Title
Abstract			
Abstract	2	Provide a structured summary that highlights context, key methods, results, and alternative analyses	Abstract
Introduction			
Background and objectives	3	Give the context for the study, the study question, and its practical relevance for decision making in policy or practice	Introduction
Methods			
Health economic analysis plan	4	Indicate whether a health economic analysis plan was developed and where available	*Not reported, as no HEAP was developed.*
Study population	5	Describe characteristics of the study population (such as age range, demographics, socioeconomic, or clinical characteristics)	Methods - Cost effectiveness model
Setting and location	6	Provide relevant contextual information that may influence findings	Methods -Intervention and cost effectiveness model
Comparators	7	Describe the interventions or strategies being compared and why chosen	Methods -Intervention
Perspective	8	State the perspective(s) adopted by the study and why chosen	Methods - Cost effectiveness model
Time horizon	9	State the time horizon for the study and why appropriate	Methods - Cost effectiveness model
Discount rate	10	Report the discount rate(s) and reason chosen	Methods - Cost effectiveness model
Selection of outcomes	11	Describe what outcomes were used as the measure(s) of benefit(s) and harm(s)	Methods - Cost effectiveness model
Measurement of outcomes	12	Describe how outcomes used to capture benefit(s) and harm(s) were measured	Methods – Input parameters – Utilities

Section/topic	Item No	Guidance for reporting	Reported in section
Valuation of outcomes	13	Describe the population and methods used to measure and value outcomes	Methods – Input parameters – Utilities
Measurement and valuation of resources and costs	14	Describe how costs were valued	Methods – Input parameters – Costs
Currency, price date, and conversion	15	Report the dates of the estimated resource quantities and unit costs, plus the currency and year of conversion	Methods – Input parameters – Costs
Rationale and description of model	16	If modelling is used, describe in detail and why used. Report if the model is publicly available and where it can be accessed	Methods – Cost effectiveness model
Analytics and assumptions	17	Describe any methods for analysing or statistically transforming data, any extrapolation methods, and approaches for validating any model used	Methods – Analysis – Cost-effectiveness analysis
Characterizing heterogeneity	18	Describe any methods used for estimating how the results of the study vary for subgroups	Methods – Analysis – Cost-effectiveness analysis
Characterizing distributional effects	19	Describe how impacts are distributed across different individuals or adjustments made to reflect priority populations	*Not included in the cost-effectiveness analysis, but in the budget impact analysis*<br><br>Methods – Analysis – BI model
Characterizing uncertainty	20	Describe methods to characterise any sources of uncertainty in the analysis	Methods – Analysis – Cost-effectiveness analysis
Approach to engagement with patients and others affected by the study	21	Describe any approaches to engage patients or service recipients, the general public, communities, or stakeholders (such as clinicians or payers) in the design of the study	*Not reported, as no engagement with stakeholders was included in the analysis.*
Results			
Study parameters	22	Report all analytic inputs (such as values, ranges, references) including uncertainty or distributional assumptions	Tables 1 and 2
Summary of main results	23	Report the mean values for the main categories of costs and outcomes of interest and summarise them in the most appropriate overall measure	Table 3
Effect of uncertainty	24	Describe how uncertainty about analytic judgments, inputs, or projections affect findings. Report the effect of choice of discount rate and time horizon, if applicable	Table 3, figure 3
Effect of engagement with patients and others affected by the study	25	Report on any difference patient/service recipient, general public, community, or stakeholder involvement made to the approach or findings of the study	*Not reported, as no engagement with stakeholders was included in the analysis.*
Discussion			
Study findings, limitations, generalizability, and current knowledge	26	Report key findings, limitations, ethical or equity considerations not captured, and how these could affect patients, policy, or practice	Discussion

Section/topic	Item No	Guidance for reporting	Reported in section
Other relevant information			
Source of funding	27	Describe how the study was funded and any role of the funder in the identification, design, conduct, and reporting of the analysis	Title page - Statements and declarations
Conflicts of interest	28	Report authors conflicts of interest according to journal or International Committee of Medical Journal Editors requirements	Title page - Competing interests

Key parameters budget impact analysis

Input	Group			Reference
Eligible population	12-59 years old	60-64 years old	65 years old and older	
Total population size in 2022 (millions)	10.7	1.2	3.5	[2]
Percentage of people at high-risk for influenza-related complications (base case)	8.9%	30.8%	50.7%	[3]
Vaccination coverage high-risk groups (base case)	32.0%	53.0%	70.6%	[3]
Vaccination coverage best-case scenario, based on COVID-19 booster campaign	50.0%	75.0%	80.0%	[4]
Vaccination coverage normal-risk groups	Not included	29.5%	51.8%	[3]
Percentage of patients consulting within 48 hours after symptom onset	67%			[5]
Percentage of patients consulting with true influenza	38%			[6]
Influenza-like illness consults during reference seasons (total per 100,000 population)	5-14 years old	15-64 years old	65 years old and older	
2013-2014	983	1016	1507	[7]
2014-2015	2096	2180	3186	
2015-2016	1911	1566	2123	
2016-2017	1512	1416	2543	
2017-2018	1891	2033	2977	
2018-2019	1222	1267	1867	
Start month influenza season	October			Assumed
End month influenza season	May			Assumed
Complications	Baloxavir arm	Placebo arm		
Hospitalization	0.8%	1.3%		[8]
Sinusitis	0.3%	2.1%		
Otitis media	0%	0.8%		
Bronchitis	1.8%	6.0%		
Outpatient pneumonia	0%	0.8%		
No complication	97.1%	89.0%		
Costs	Total population			
Baloxavir, including pharmacy fee	€133			[9]
Antibiotic treatment, including pharmacy fee	€18.70			[9]
GP consult	€34.36			[10]
Hospitalization	€7,340.07			[11]
Outpatient pneumonia	€647.46			[12]

Cost-effectiveness plane base-case analysis

Displayed here are 10,000 model replications

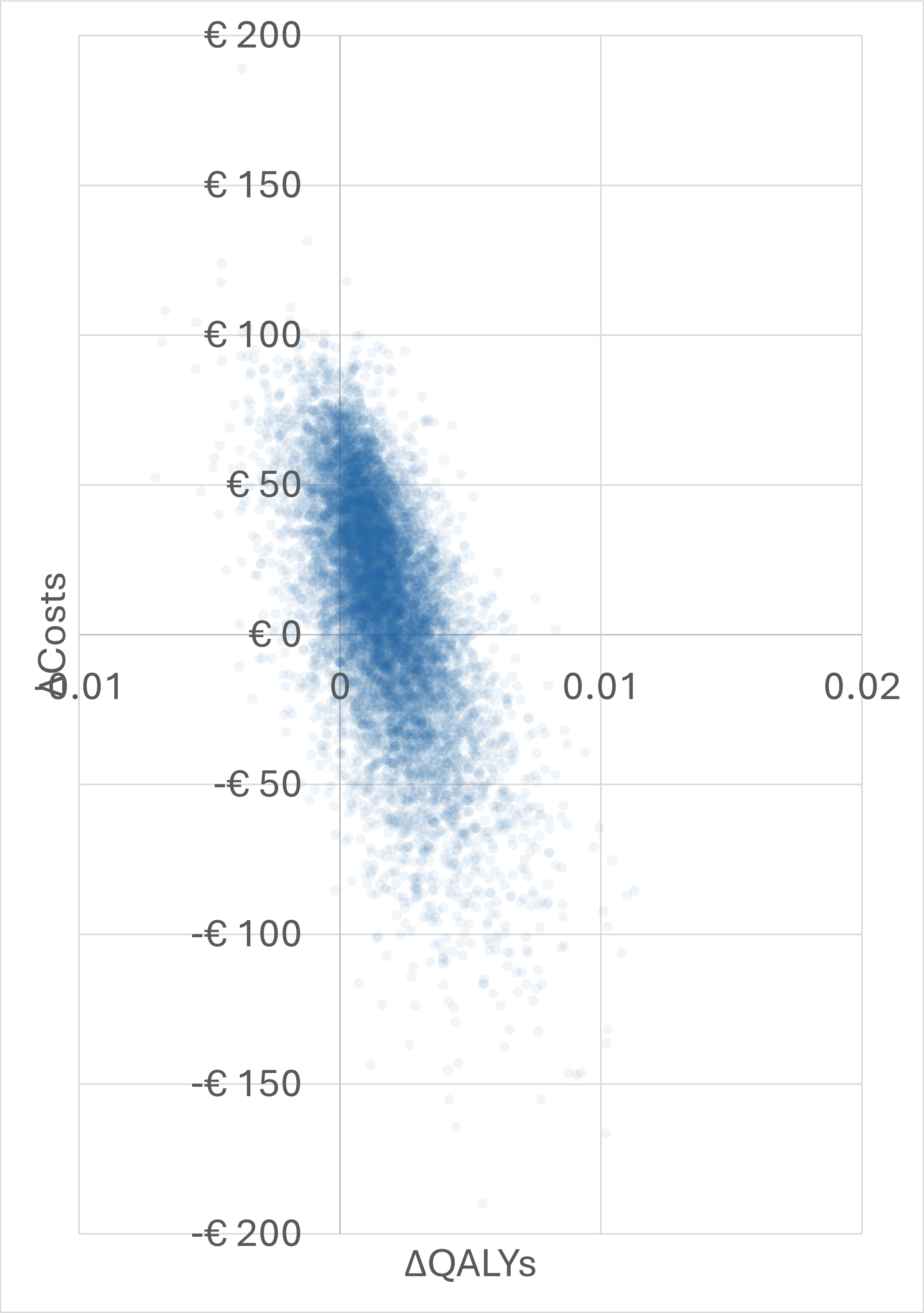

Extended results scenario analyses cost-effectiveness

Scenarios are based on 1,000 model replications.

Scenario 1 (base case)

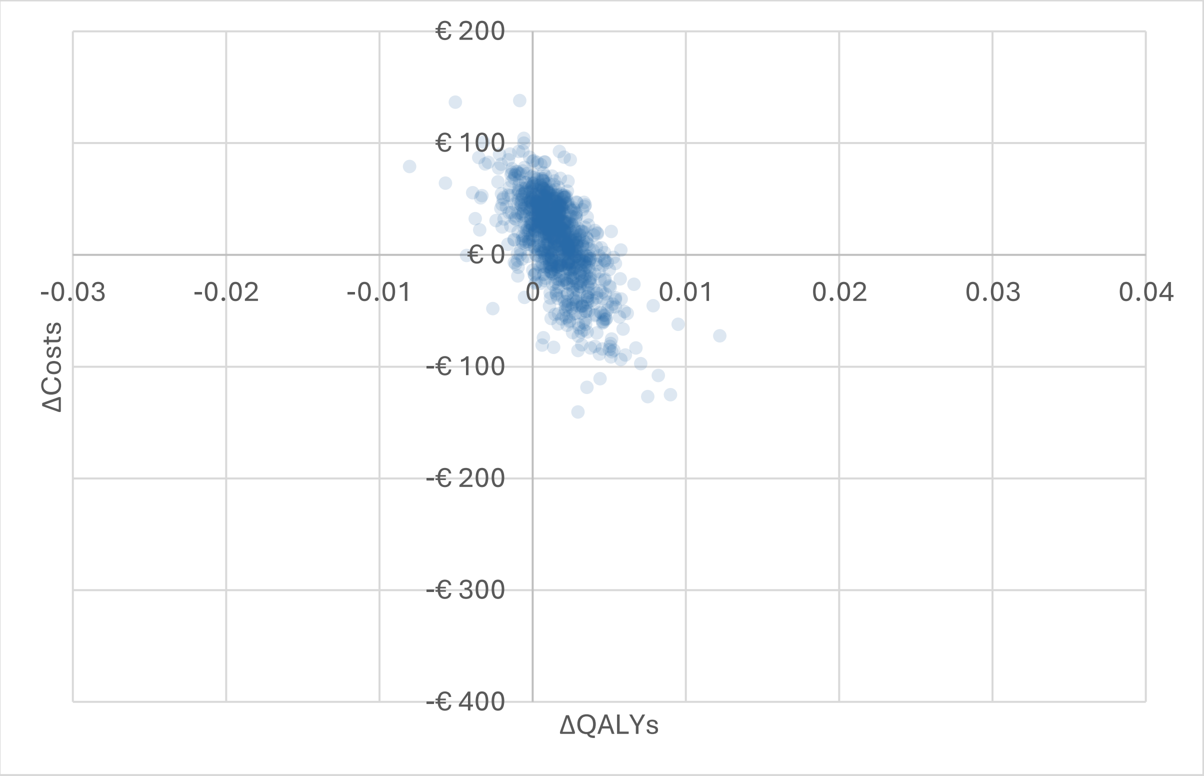

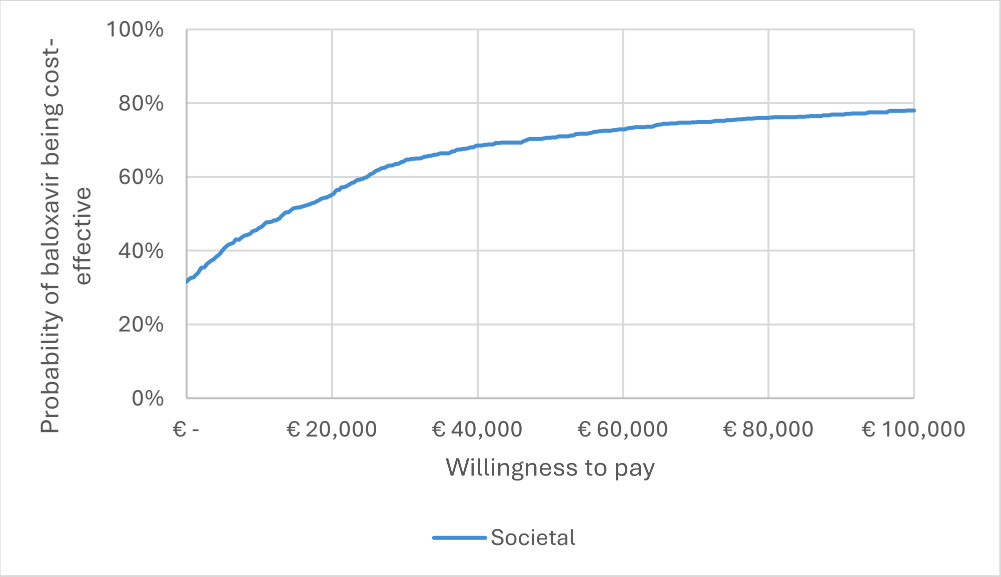

Scenario 2

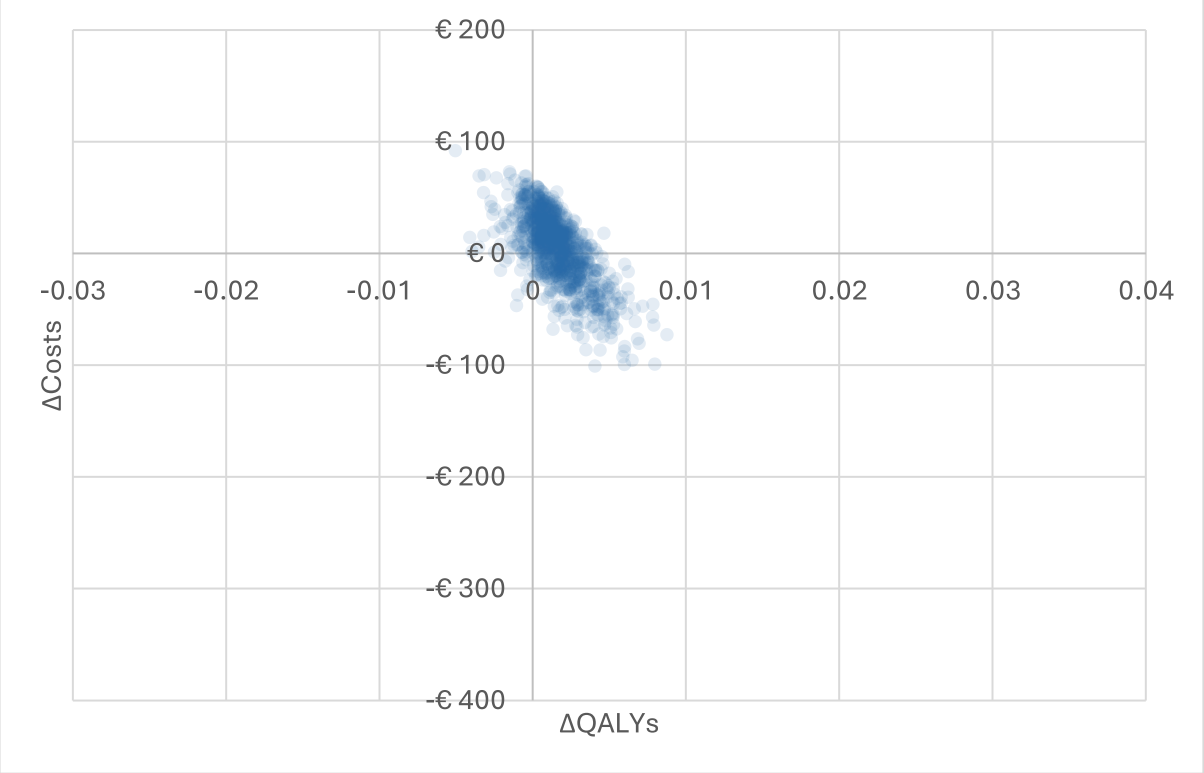

€ 200
€ 100
€ 0
-€ 100
-€ 200
-€ 300
-€ 400
-0.03
-0.02
-0.01
0
0.01
0.02
0.03
0.04
ΔCosts
ΔQALYs

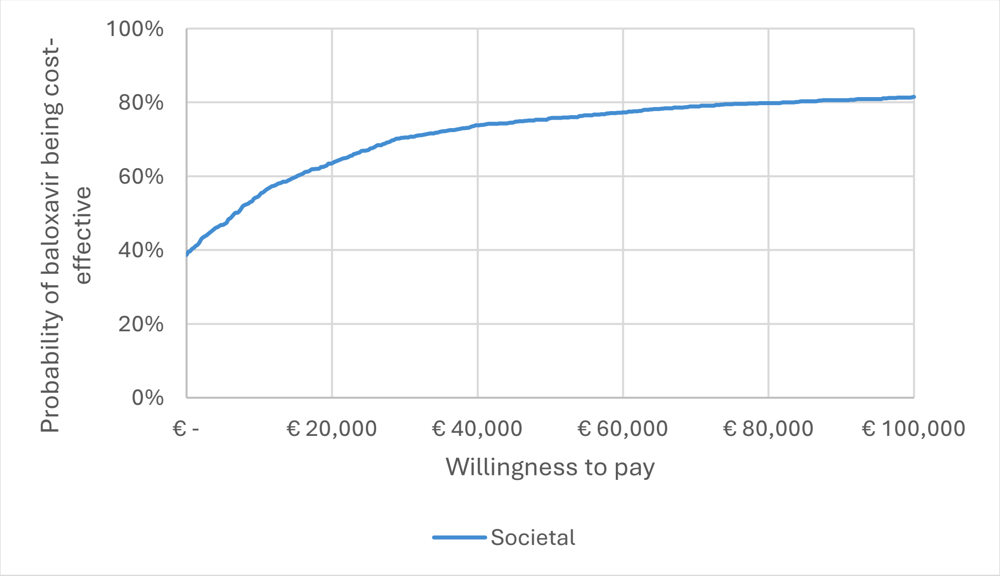

Probability of baloxavir being cost-effective
100%
80%
60%
40%
20%
0%
€ -
€ 20,000
€ 40,000
€ 60,000
€ 80,000
€ 100,000
Willingness to pay
Societal

Scenario 3

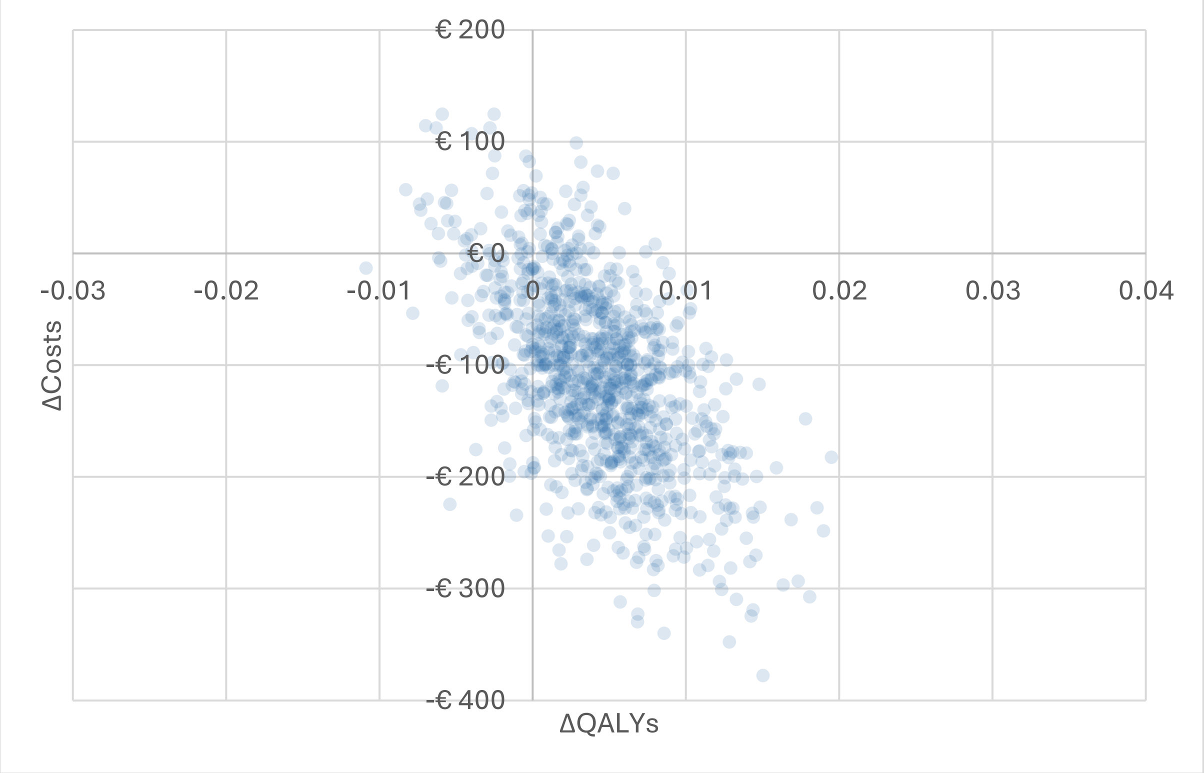
€ 200
€ 100
€ 0
-€ 100
-€ 200
-€ 300
-€ 400
-0.03
-0.02
-0.01
0
0.01
0.02
0.03
0.04
ΔCosts
ΔQALYs

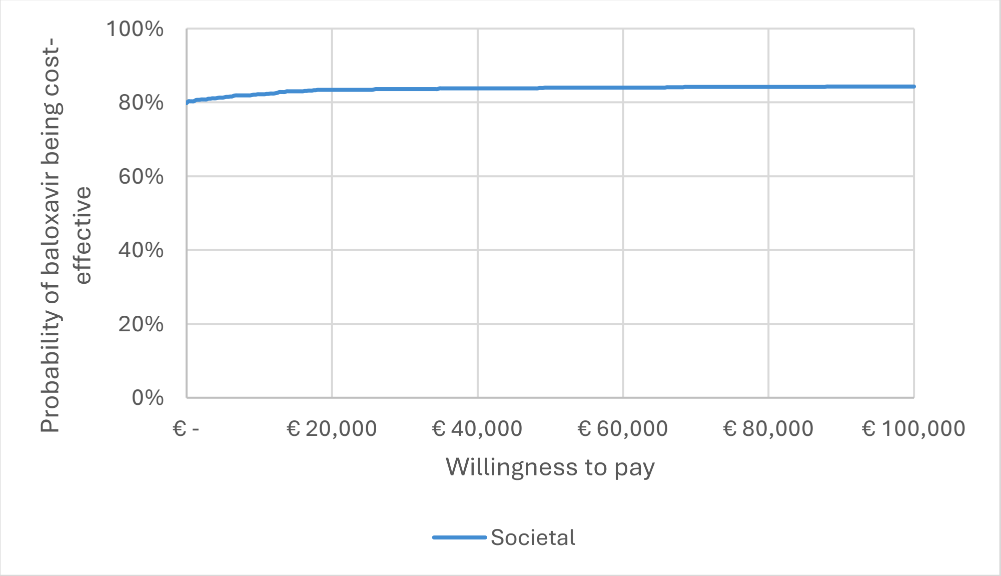
100%
80%
60%
40%
20%
0%
Probability of baloxavir being cost-effective
€ -
€ 20,000
€ 40,000
€ 60,000
€ 80,000
€ 100,000
Willingness to pay
Societal

Scenario 4

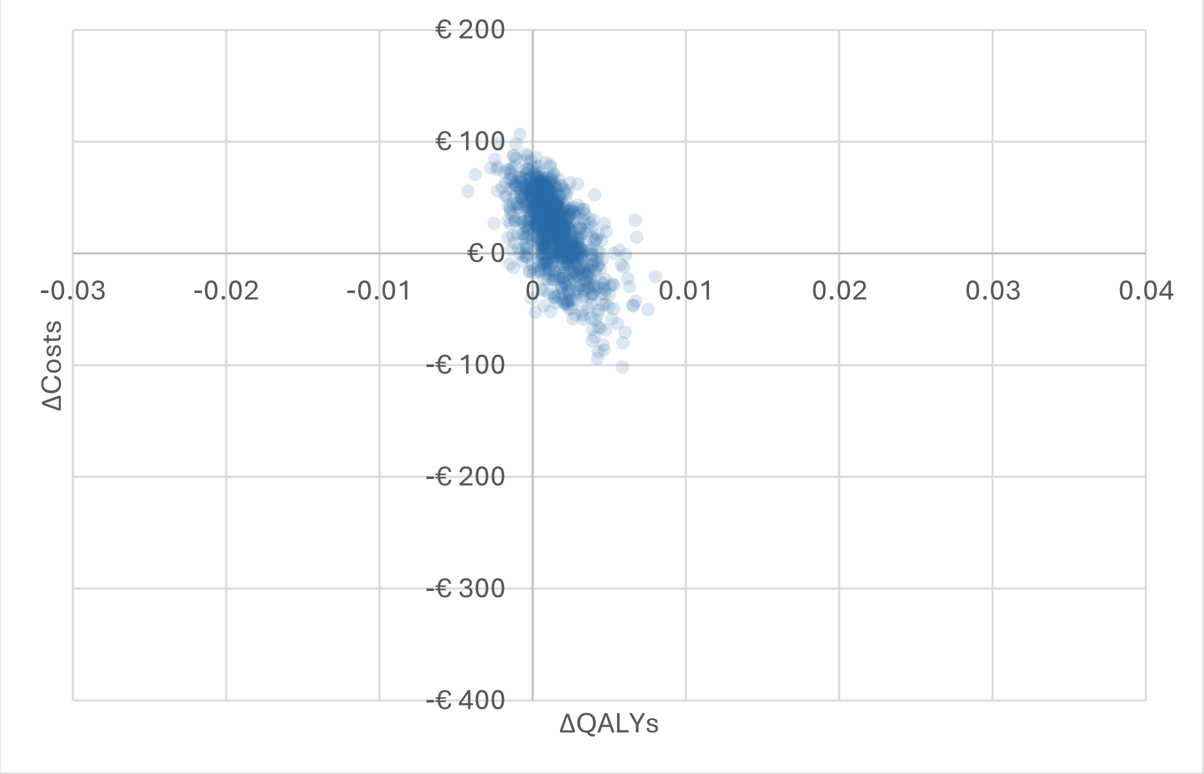
€ 200
€ 100
€ 0
-€ 100
-€ 200
-€ 300
-€ 400
-0.03
-0.02
-0.01
0
0.01
0.02
0.03
0.04
ΔCosts
ΔQALYs

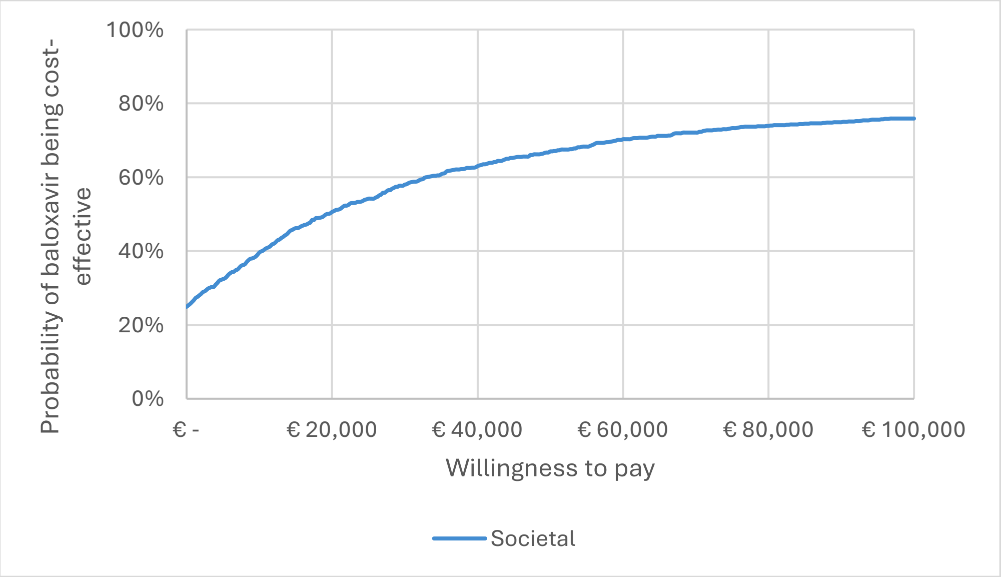
100%
80%
60%
40%
20%
0%
Probability of baloxavir being cost-effective
€ -
€ 20,000
€ 40,000
€ 60,000
€ 80,000
€ 100,000
Willingness to pay
Societal

Scenario 5

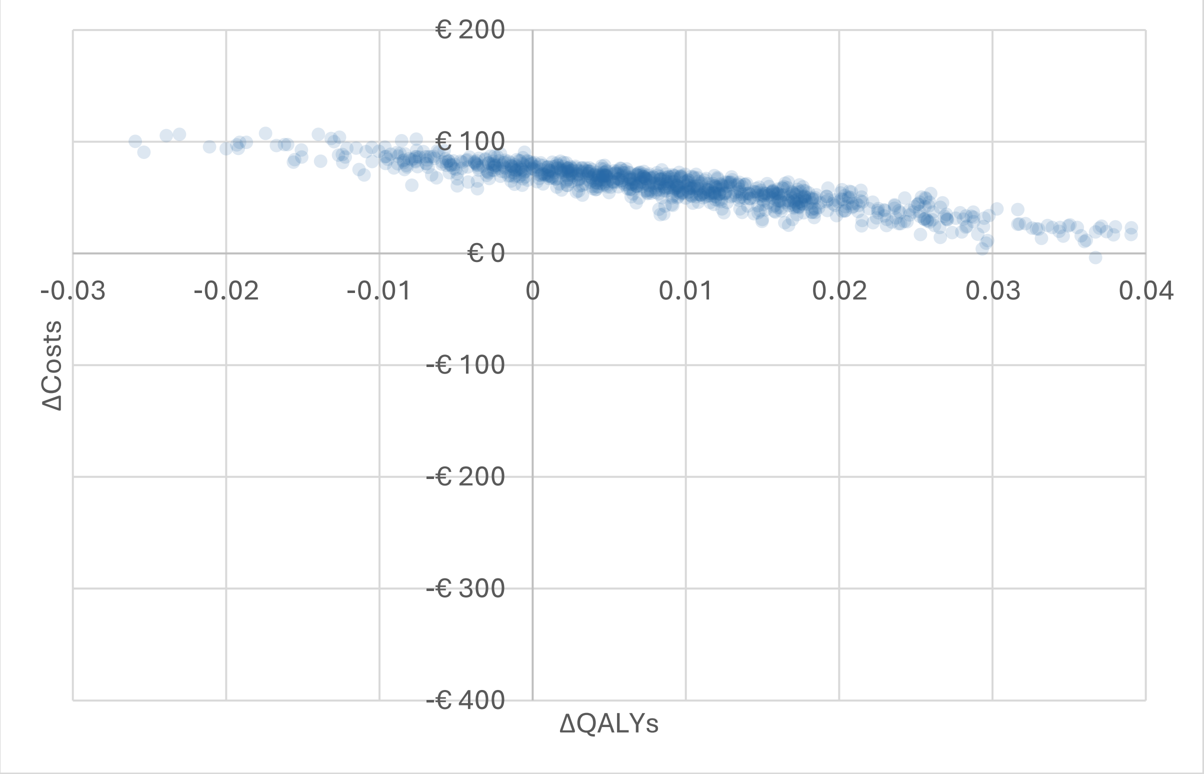
€ 200
€ 100
€ 0
-€ 100
-€ 200
-€ 300
-€ 400
-0.03
-0.02
-0.01
0
0.01
0.02
0.03
0.04
ΔCosts
ΔQALYs

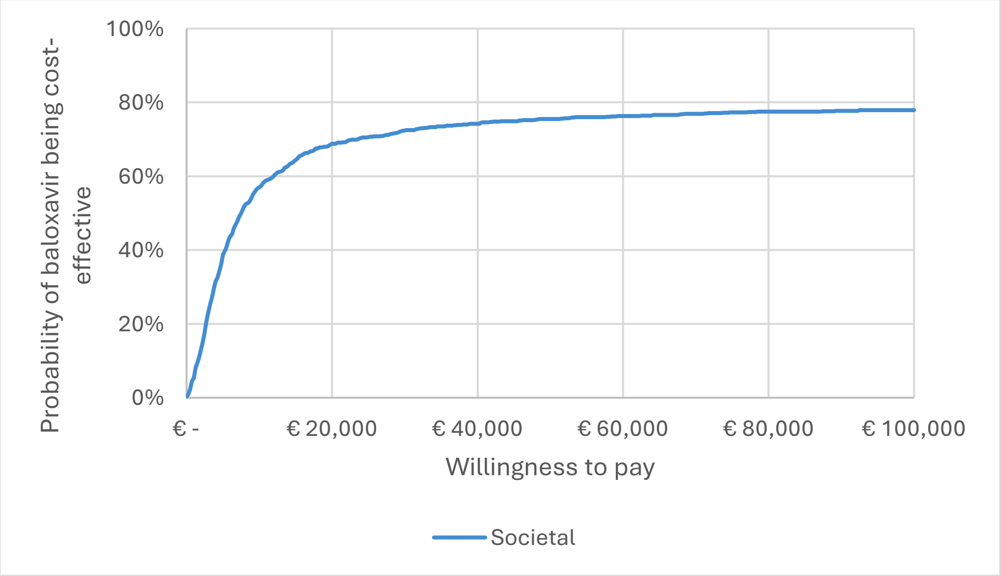
100%
80%
60%
40%
20%
0%
Probability of baloxavir being cost-effective
€ -
€ 20,000
€ 40,000
€ 60,000
€ 80,000
€ 100,000
Willingness to pay
Societal

Scenario 6

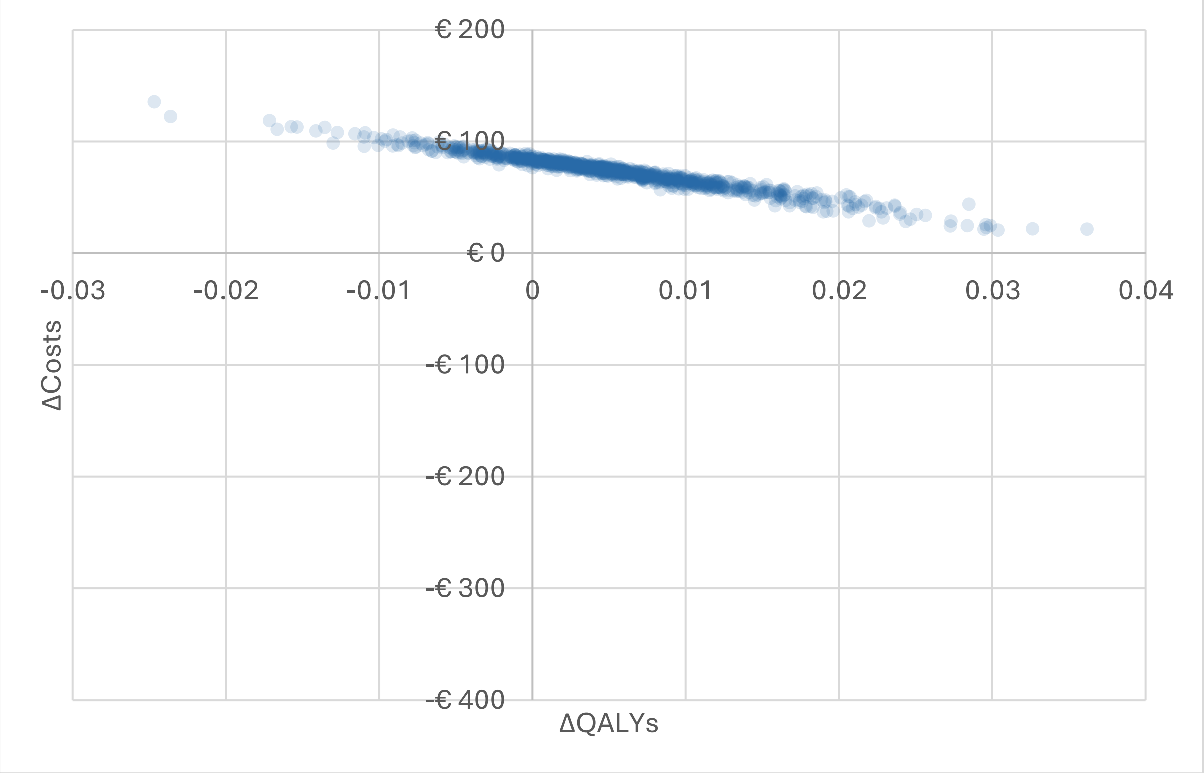

€ 200
€ 100
€ 0
-0.03
-0.02
-0.01
0
0.01
0.02
0.03
0.04
ΔCosts
-€ 100
-€ 200
-€ 300
-€ 400
ΔQALYs

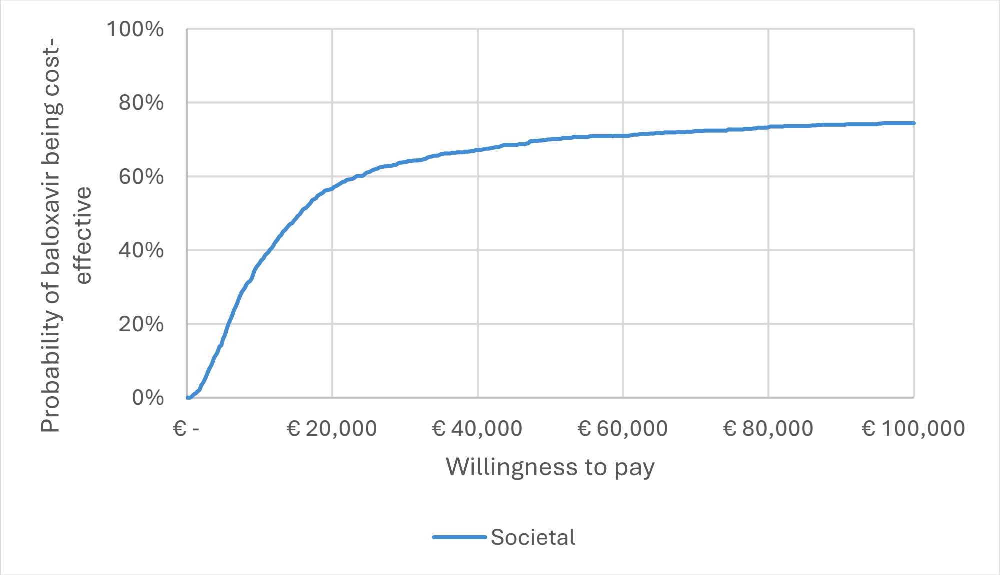

Probability of baloxavir being cost-effective
100%
80%
60%
40%
20%
0%
€ -
€ 20,000
€ 40,000
€ 60,000
€ 80,000
€ 100,000
Willingness to pay
Societal

Scenario 7

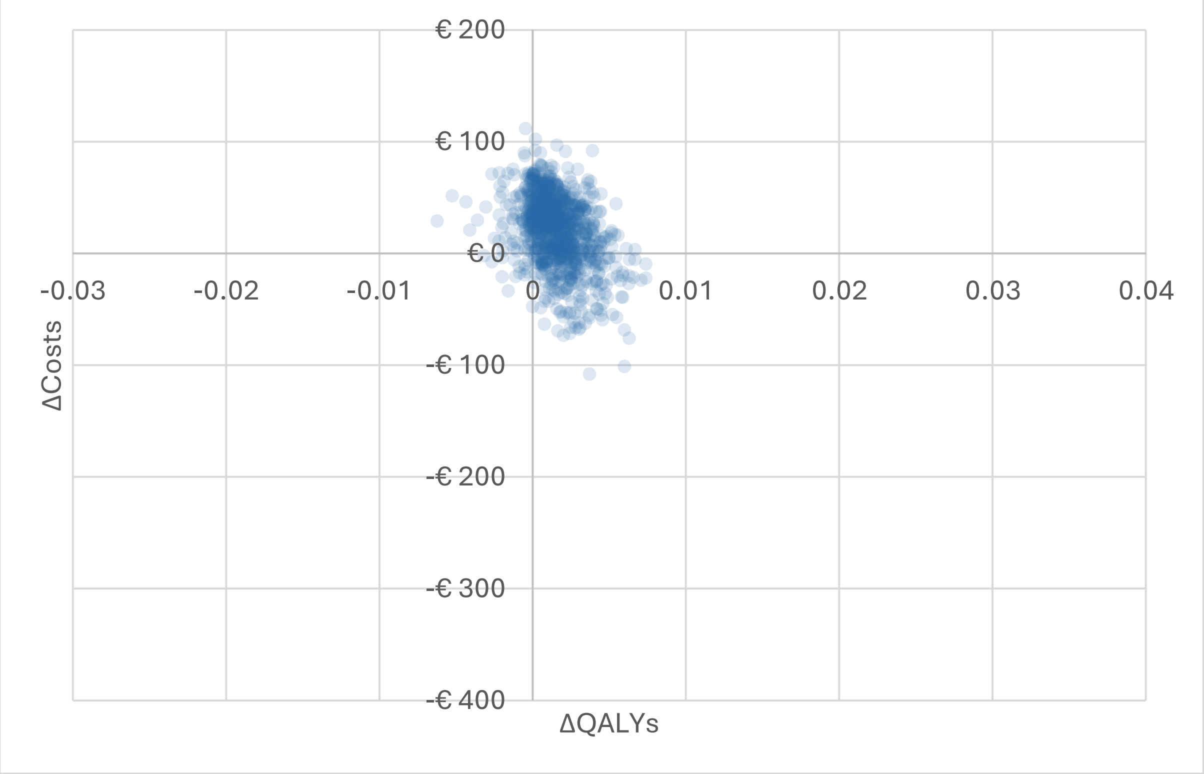
€ 200
€ 100
€ 0
-€ 100
-€ 200
-€ 300
-€ 400
-0.03
-0.02
-0.01
0
0.01
0.02
0.03
0.04
ΔCosts
ΔQALYs

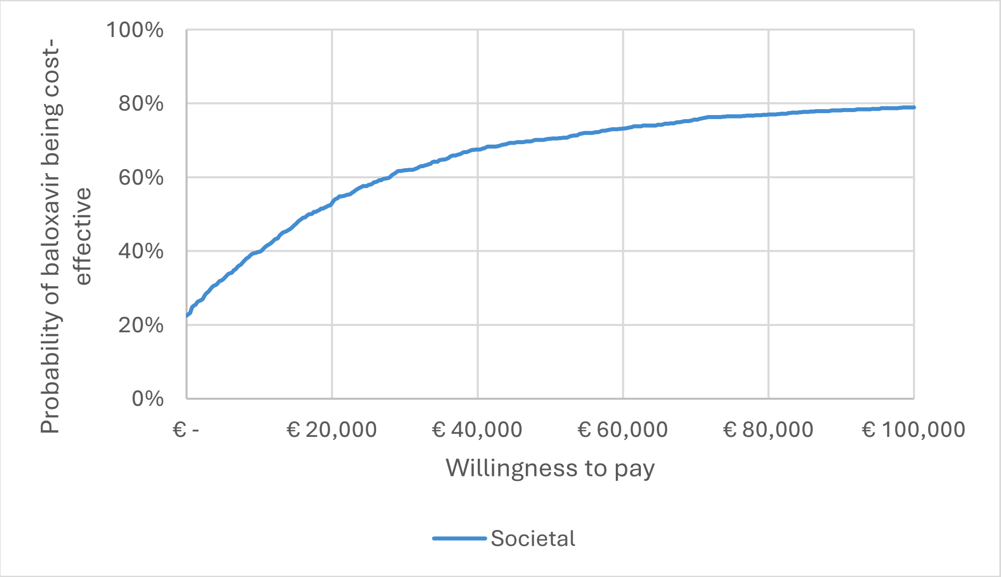
Probability of baloxavir being cost-effective
100%
80%
60%
40%
20%
0%
€ -
€ 20,000
€ 40,000
€ 60,000
€ 80,000
€ 100,000
Willingness to pay
Societal

Scenario 8

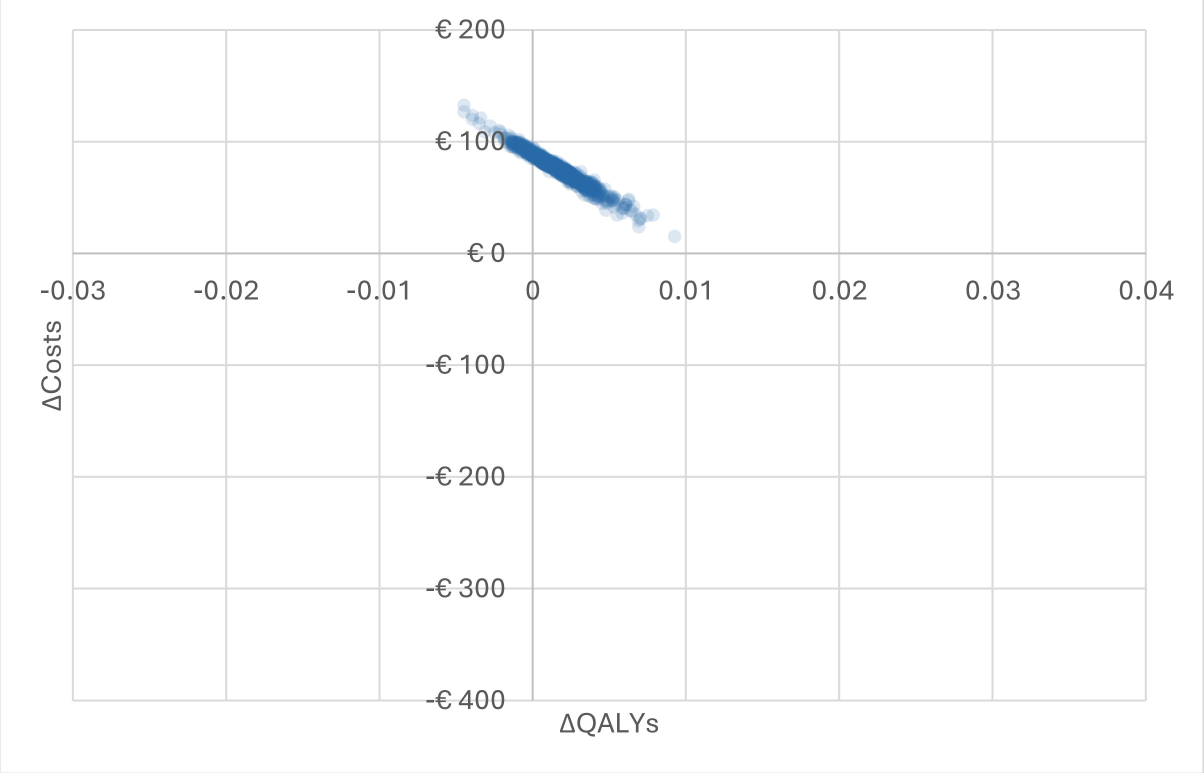
€ 200
€ 100
€ 0
-0.03
-0.02
-0.01
0
0.01
0.02
0.03
0.04
-€ 100
-€ 200
-€ 300
-€ 400
ΔCosts
ΔQALYs

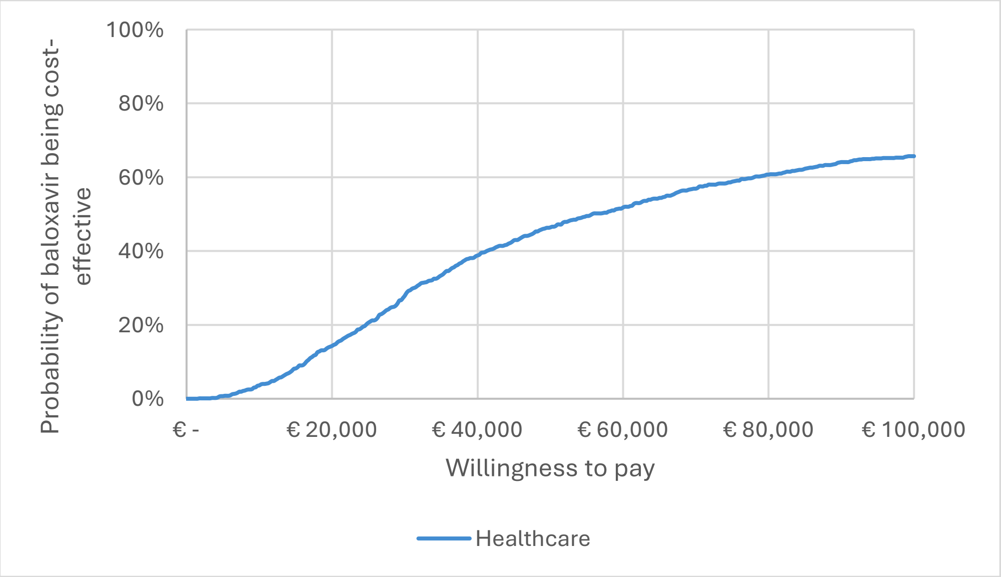
Probability of baloxavir being cost-effective
100%
80%
60%
40%
20%
0%
€ -
€ 20,000
€ 40,000
€ 60,000
€ 80,000
€ 100,000
Willingness to pay
Healthcare

Scenario 9

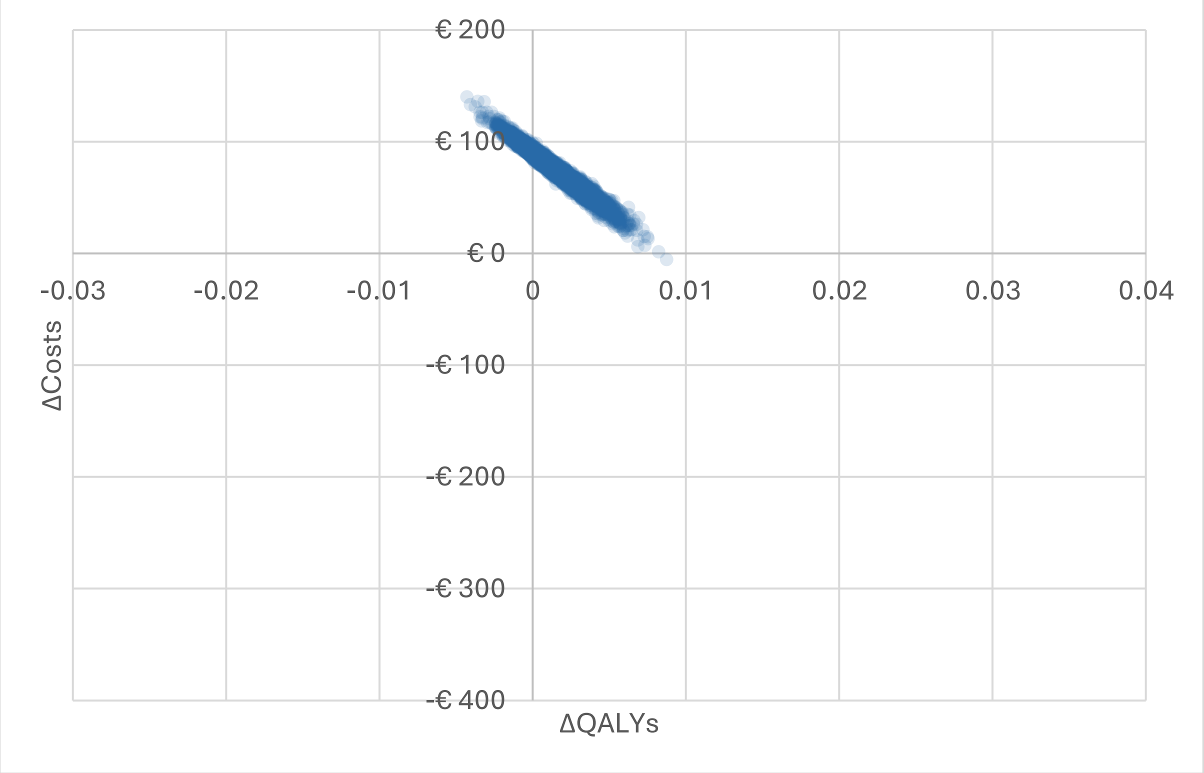
€ 200
€ 100
€ 0
-0.03
-0.02
-0.01
0
0.01
0.02
0.03
0.04
ΔCosts
-€ 100
-€ 200
-€ 300
-€ 400
ΔQALYs

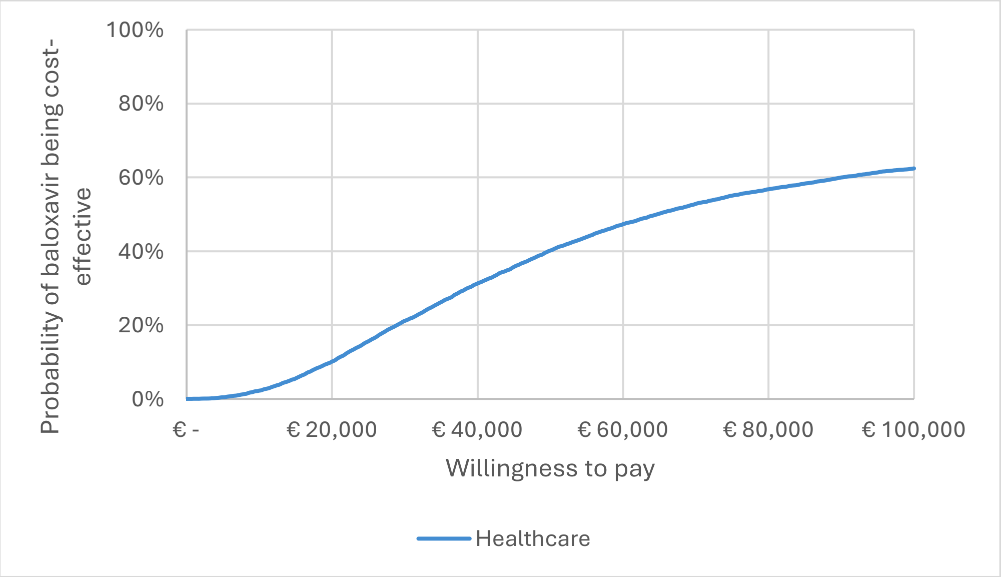
Probability of baloxavir being cost-effective
100%
80%
60%
40%
20%
0%
€ -
€ 20,000
€ 40,000
€ 60,000
€ 80,000
€ 100,000
Willingness to pay
Healthcare

References

1. Husereau, D., Drummond, M., Augustovski, F., de Bekker-Grob, E., Briggs, A.H., Carswell, C., Caulley, L., Chaiyakunapruk, N., Greenberg, D., Loder, E., Mauskopf, J., Mullins, C.D., Petrou, S., Pwu, R.-F., Staniszewska, S.: Consolidated Health Economic Evaluation Reporting Standards 2022 (CHEERS 2022) statement: updated reporting guidance for health economic evaluations. Eur J Health Econ. 23, 1309–1317 (2022). https://doi.org/10.1007/s10198-021-01426-6
2. Statistics Netherlands: CBS StatLine - Bevolking; geslacht, leeftijd en burgerlijke staat, 1 januari, http://statline.cbs.nl/Statweb/publication/?DM=SLNL&PA=7461BEV&D1=0&D2=1-2&D3=1-100&D4=61&HDR=T,G3&STB=G1,G2&VW=T
3. Heins, M., Hooiveld, Mari??tte, Korevaar, J., Nederlands instituut voor onderzoek van de gezondheidszorg (Utrecht), Rijksinstituut voor Volksgezondheid en Milieu (Bilthoven): Monitor Vaccinatiegraad Nationaal Programma Grieppreventie 2019. NIVEL, Utrecht (2020)
4. Cijfers COVID-19 vaccinatieprogramma | RIVM, https://www.rivm.nl/covid-19-vaccinatie/cijfers-vaccinatieprogramma
5. Butler, C.C., van der Velden, A.W., Bongard, E., Saville, B.R., Holmes, J., Coenen, S., Cook, J., Francis, N.A., Lewis, R.J., Godycki-Cwirko, M., Llor, C., Chlabicz, S., Lionis, C., Seifert, B., Sundvall, P.-D., Colliers, A., Aabenhus, R., Bjerrum, L., Jonassen Harbin, N., Lindbæk, M., Glinz, D., Bucher, H.C., Kovács, B., Radzeviciene Jurgute, R., Touboul Lundgren, P., Little, P., Murphy, A.W., De Sutter, A., Openshaw, P., de Jong, M.D., Connor, J.T., Matheeussen, V., Ieven, M., Goossens, H., Verheij, T.J.: Oseltamivir plus usual care versus usual care for influenza-like illness in primary care: an open-label, pragmatic, randomised controlled trial. The Lancet. 395, 42–52 (2020). https://doi.org/10.1016/S0140-6736(19)32982-4
6. Dolk, F.C.K., de Boer, P.T., Nagy, L., Donker, G.A., Meijer, A., Postma, M.J., Pitman, R.: Consultations for Influenza-Like Illness in Primary Care in The Netherlands: A Regression Approach. Value in Health. 24, 11–18 (2021). https://doi.org/10.1016/j.jval.2020.10.013
7. World Health Organization: WHO FLUMART OUTPUTS, https://apps.who.int/flumart/Default?ReportNo=16
8. Ison, M.G., Portsmouth, S., Yoshida, Y., Shishido, T., Mitchener, M., Tsuchiya, K., Uehara, T., Hayden, F.G.: Early treatment with baloxavir marboxil in high-risk adolescent and adult outpatients with uncomplicated influenza (CAPSTONE-2): a randomised, placebo-controlled, phase 3 trial. The Lancet Infectious Diseases. 20, 1204–1214 (2020). https://doi.org/10.1016/S1473-3099(20)30004-9
9. Zorginstituut Nederland: Medicijnkosten, https://www.medicijnkosten.nl/
10. Hakkaart-van Roijen, L., Van der Linden, N., Bouwmans, C., Kanters, T., Tan, S.: Kostenhandleiding: methodologie van kostenonderzoek en referentieprijzen voor economische evaluaties in de gezondheidszorg. Zorginstituut Nederland, Diemen (2015)
11. Marbus, S.D., Schweitzer, V.A., Groeneveld, G.H., Oosterheert, J.J., Schneeberger, P.M., van der Hoek, W., van Dissel, J.T., van Gageldonk-Lafeber, A.B., Mangen, M.-J.: Incidence and costs of hospitalized adult influenza patients in The Netherlands: a retrospective observational study. Eur J Health Econ. 21, 775–785 (2020). https://doi.org/10.1007/s10198-020-01172-1
12. Fens, T., van der Pol, S., Kocks, J.W.H., Postma, M.J., van Boven, J.F.M.: Economic Impact of Reducing Inappropriate Inhaled Corticosteroids Use in Patients With Chronic Obstructive Pulmonary Disease: ISPOR's Guidance on Budget Impact in Practice. Value in Health. 22, 1092–1101 (2019). https://doi.org/10.1016/j.jval.2019.05.006
